# Activation of SARS-CoV-2 neutralizing antibody is slower than elevation of spike-specific IgG, IgM, and nucleocapsid-specific IgG antibodies

**DOI:** 10.1038/s41598-022-19073-z

**Published:** 2022-09-01

**Authors:** Maika Takahashi, Tomohiko Ai, Konomi Sinozuka, Yuna Baba, Gene Igawa, Shuko Nojiri, Takamasa Yamamoto, Maiko Yuri, Satomi Takei, Kaori Saito, Yuki Horiuchi, Takayuki Kanno, Minoru Tobiume, Abdullah Khasawneh, Faith Jessica Paran, Makoto Hiki, Mitsuru Wakita, Takashi Miida, Tadaki Suzuki, Atsushi Okuzawa, Kazuhisa Takahashi, Toshio Naito, Yoko Tabe

**Affiliations:** 1 Department of Clinical Laboratory, Juntendo University Hospital, Tokyo, Japan; 2 Department of Clinical Laboratory Medicine, Juntendo University Graduate School of Medicine, Hongo 2-1-2, Bunkyo-ku, Tokyo, 113-8421 Japan; 3 Medical Technology Innovation Center, Juntendo University, Tokyo, Japan; 4 Department of Pathology, National Institute of Infectious Diseases, Tokyo, Japan; 5 Department of Research Support Utilizing Bioresource Bank, Juntendo University Graduate School of Medicine, Tokyo, Japan; 6 Department of Emergency Medicine, Juntendo University Faculty of Medicine, Tokyo, Japan; 7 Department of Cardiovascular Biology and Medicine, Juntendo University Faculty of Medicine, Tokyo, Japan; 8 Department of Respiratory Medicine, Juntendo University Graduate School of Medicine, Tokyo, Japan; 9 Department of General Medicine, Juntendo University Graduate School of Medicine, Tokyo, Japan

**Keywords:** Laboratory techniques and procedures, Viral infection

## Abstract

COVID-19 antibody testing has been developed to investigate humoral immune response in SARS-CoV-2 infection. To assess the serological dynamics and neutralizing potency following SARS-CoV-2 infection, we investigated the neutralizing (NT) antibody, anti-spike, and anti-nucleocapsid antibodies responses using a total of 168 samples obtained from 68 SARS-CoV-2 infected patients. Antibodies were measured using an authentic virus neutralization assay, the high-throughput laboratory measurements of the Abbott Alinity quantitative anti-spike receptor-binding domain IgG (S-IgG), semiquantitative anti-spike IgM (S-IgM), and anti-nucleocapsid IgG (N-IgG) assays. The quantitative measurement of S-IgG antibodies was well correlated with the neutralizing activity detected by the neutralization assay (r = 0.8943, p < 0.0001). However, the kinetics of the SARS-CoV-2 NT antibody in severe cases were slower than that of anti-S and anti-N specific antibodies. These findings indicate a limitation of using the S-IgG antibody titer, detected by the chemiluminescent immunoassay, as a direct quantitative marker of neutralizing activity capacity. Antibody testing should be carefully interpreted when utilized as a marker for serological responses to facilitate diagnostic, therapeutic, and prophylactic interventions.

## Introduction

Coronavirus disease 2019 (COVID-19), caused by severe acute respiratory syndrome coronavirus 2 (SARS-CoV-2), is a major public health concern. The reverse transcription polymerase chain reaction (RT-PCR) test is considered the gold standard for detecting the presence of viral RNA. However, the accuracy of RT-PCR relies heavily on sample collecting timing, type, storage, handling, and processing. RT-PCR products of SARS-CoV-2 nucleotides can be detected several days after onset^1^, with sensitivity declining after 1–2 weeks^2^. Conversely, the sensitivity of serological assays increases 2 weeks after symptom onset^2^, indicating combined RT-PCR and antibody testing can be complementary for laboratory diagnosis.

SARS-CoV-2 is composed of four structural proteins, spike (S), nucleocapsid (N), envelope (E), and membrane (M), and more than 20 nonstructural proteins. Of these, S and N proteins have been used as antibody assay targets^3,4^. N proteins are RNA-binding proteins consisting of nucleocapsids, which are highly immunogenic and expressed abundantly during infection. Therefore, antibodies that bind to N proteins can be indicators of exposure to the virus^5–8^. However, as N proteins are shielded within the virion, they may not be involved in the neutralization of the virus^9^.

S proteins are composed of the S1 and S2 subunits and are expressed on the surface of the virus. The S1 protein includes the N-terminal domain (NTD) and receptor-binding domain (RBD), whereas the S2 protein promotes membrane fusion^10^. The RBD is responsible for direct binding to angiotensin-converting enzyme 2 (ACE2), a host cell receptor responsible for mediating SARS-CoV-2 attachment^10^. Because the RBD is predominantly targeted by the immune system, with 90% of the neutralizing activity of SARS-CoV-2 immune serum targeting RBD^11–13^, anti RBD antibodies have the potential to neutralize viral entry into cells and are crucial in the protective immune response to SARS-CoV-2 infection^14,15^. Furthermore, NTD-specific antibodies have also been reported to neutralize SARS-CoV-2^16^. These findings indicate that not only RBD, used as the current target of vaccines, but also NTD could be an attractive target for vaccine design.

With respect to the kinetics of neutralizing and anti-S protein antibodies in COVID-19 patients, the titers of both neutralizing and anti-S protein antibodies have been shown to be higher in symptomatic patients than in asymptomatic patients^11,17^. While coordinated antibody responses with CD4+ T cell and CD8+ T cell are protective, uncoordinated responses fail to combat disease and show impaired immune responses to SARS-CoV-2^18^. These findings indicate that humoral adaptive immune responses are stronger in critically ill patients, but uncontrolled immune responses may be involved in the progression of the pathology. One study, using an anti-RBD IgG neutralization potency index (NT50/IgG), showed that deceased patients had higher levels of anti-RBD IgG antibodies with significantly lower neutralization potency, suggesting the higher levels of anti-RBD IgG antibodies in critical patients did not contribute to neutralization^19^. Considering the discussed studies, although antibody responses represent key immune correlates of protection and recovery for SARS-CoV-2, further analysis of the association of the kinetics between anti-RBD IgG and neutralizing antibodies is required.

In this study, an authentic virus neutralization assay was used to investigate serological kinetics and neutralization potential after SARS-CoV-2 infection^20^. The clinical performance of high-throughput, widely available laboratory measurements of the three serological assays for SARS-CoV-2 antibodies was then evaluated in comparison to neutralizing activity. The assays evaluated were S protein RBD-specific IgG antibody quantification reagents (S-IgG, anti-S Abbott SARS-CoV-2 IgG II Quant), whole S protein-specific IgM antibody semi-quantitative reagents (S-IgM, anti-S Abbott Alinity SARS-CoV-2 IgM), and semi-quantitative reagents for N protein-specific IgG antibody (N-IgG, anti-N Abbott Alinity SARS-CoV-2 IgG) and analyze how neutralizing antibody function evolves during infection and promote recovery.

## Results

### Correlations between S-IgG, S-IgM, and N-IgG antibody levels and NT antibody activity

To assess the potential utility of the tested serological assays, SARS-CoV-2 IgG II Quant assay (S-IgG), anti-S SARS-CoV-2 IgM assay (S-IgM), and anti-N SARS-CoV-2 IgG assay (N-IgG), we first validated their clinical specificity and linearity. The specificities of S-IgG, S-IgM, and N-IgG were evaluated using the samples collected before the COVID-19 pandemic. Clinical linearity was examined for the quantitative S-IgG assay using five COVID-19 patient samples with elevated S-IgG antibody values. We evaluated the linearity of the S-IgG assay using five samples with elevated S-IgG titer. The S-IgG assay showed excellent linearities up to the samples in which antibody value was 17,742.5 AU/mL (Supplementary Fig. S1A), just below the manufacturer-recommended clinical reportable range of 20,000 AU/mL.

The correlations of S-IgG, S-IgM, and N-IgG antibody levels with NT antibody activities detected by the authentic virus neutralizing assay were investigated using 141 samples, in which these antibodies’ titers were measured simultaneously. COVID-19 cases were divided into Group M, including mild and moderate cases, and Group S, including severe and critical cases, according to the WHO criteria^21^.

**Figure 1:**
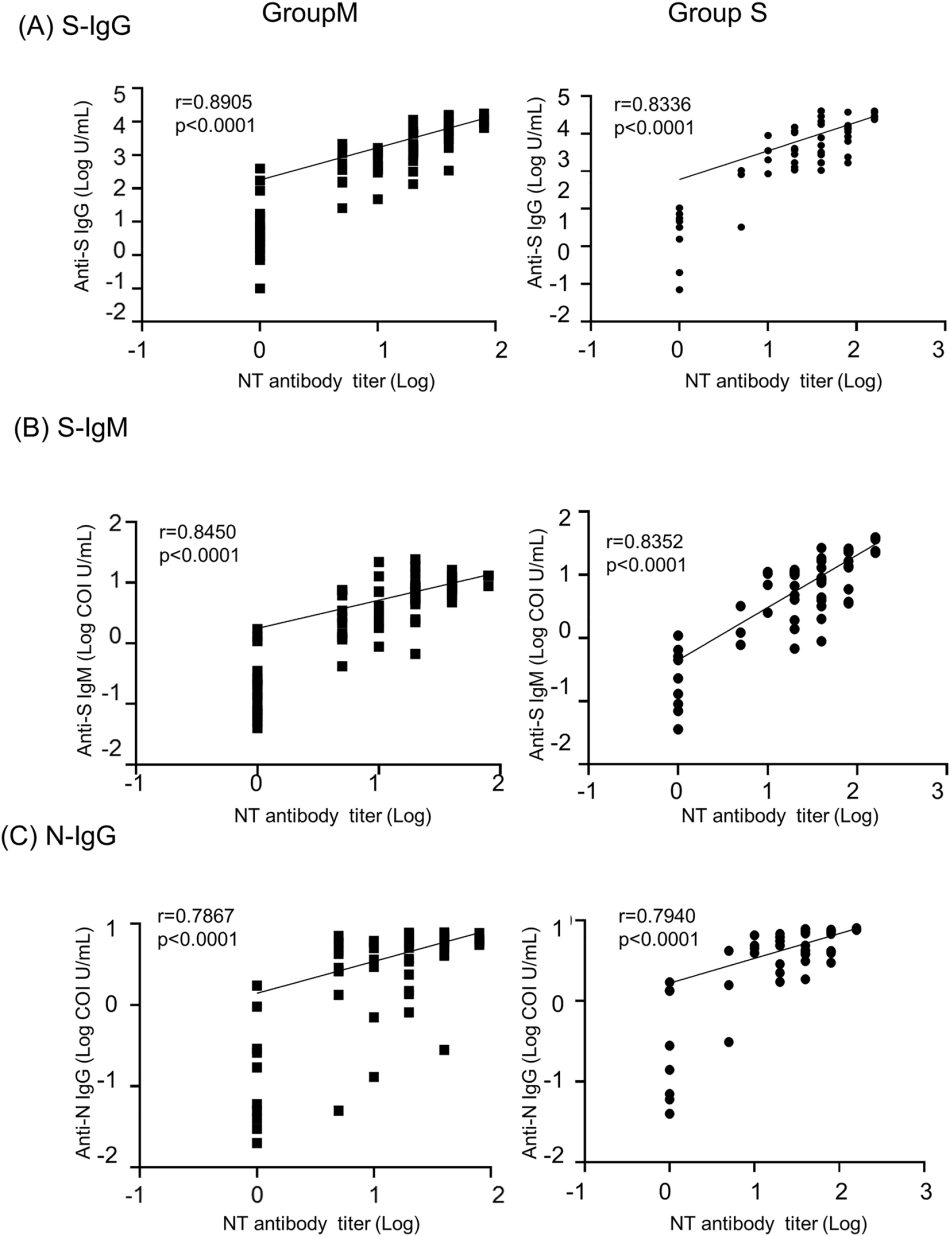
Correlations of S-IgG, S-IgM, and N-IgG assays results to NT antibody assay. (**A**) Correlation of S-IgG (anti-S SARS-CoV-2 IgG II Quant assay) and NT antibody. (**B**) Correlation of S-IgM (anti-S SARS-CoV-2 IgM assay) and NT antibody. (**C**) Correlation of N-IgG (anti-N SARS-CoV-2 IgG assay) and NT antibody. The horizontal axis and the vertical axis are logarithmic notations. One hundred forty-one samples were measured for S-IgG, S-IgM, N-IgG, and NT antibody titers and their correlations were analyzed. Black squares are Group M samples (n = 80 from 43 patients) and black circles are Group S samples (n = 61 from 14 patients).

The quantitative S-IgG assay showed a strong correlation with the NT antibody titers (Group M, r = 0.8905, p < 0.0001; Group S, r = 0.8336, p < 0.0001) (Fig. 1). The semi-quantitative S-IgM and N-IgG also showed a positive correlation with NT activity (S-IgM; Group M, r = 0.8450, p < 0.0001; Group S, r = 0.8352, p < 0.0001; N-IgG; Group M r = 0.7867, p < 0.0001; Group S, r = 0.7940, p < 0.0001). There were no clear differences in correlation between group S and group M.

As shown in Table 1, among the NT antibody negative samples (n = 39), 6 samples (15%) were positive for S-IgG, 4 (10%) were positive for S-IgM, and 3 (8%) were positive for N-IgG. Of the samples with NT antibody titers from 5 to 20 (n = 28), 4 samples (14%) were negative for S-IgG, 4 samples (14%) were negative for S-IgM, and 5 samples (18%) were negative for N-IgG. Samples with more than 20 titer of NT antibody were almost all positive for S-IgG, S-IgM, and N-IgG.

**Table 1:** Correlation between SARS-CoV-2 NT antibody activities and S-IgG, S-IgM, N-IgG antibody levels.

NT antibody (titers)	Positive/negative	Sample number	S-IgG		S-IgM		N-IgG	
			Positive (%)	Negative (%)	Positive (%)	Negative (%)	Positive (%)	Negative (%)
≧ 0, < 5	Negative	39	6 (15)	33 (85)	4 (10)	35 (90)	3 (8)	36 (92)
≧ 5, < 10	Positive	13	11 (85)	2 (15)	11 (85)	2 (15)	10 (77)	3 (23)
≧ 10, < 20	Positive	15	13 (87)	2 (13)	13 (87)	2 (13)	13 (87)	2 (13)
≧ 20, < 40	Positive	26	26 (100)	0 (0)	25 (96)	1 (4)	25 (96)	1 (4)
≧ 40, < 80	Positive	29	29 (100)	0 (0)	28 (97)	1 (3)	28 (97)	1 (3)
≧ 80, < 160	Positive	14	14 (100)	0 (0)	14 (100)	0 (0)	14 (100)	0 (0)
≦ 160	Positive	5	5 (100)	0 (0)	5 (100)	0 (0)	5 (100)	0 (0)

### Kinetics of NT antibody and S-IgG, S-IgM, and N-IgG antibodies after SARS-CoV-2 infection

**Figure 2:**
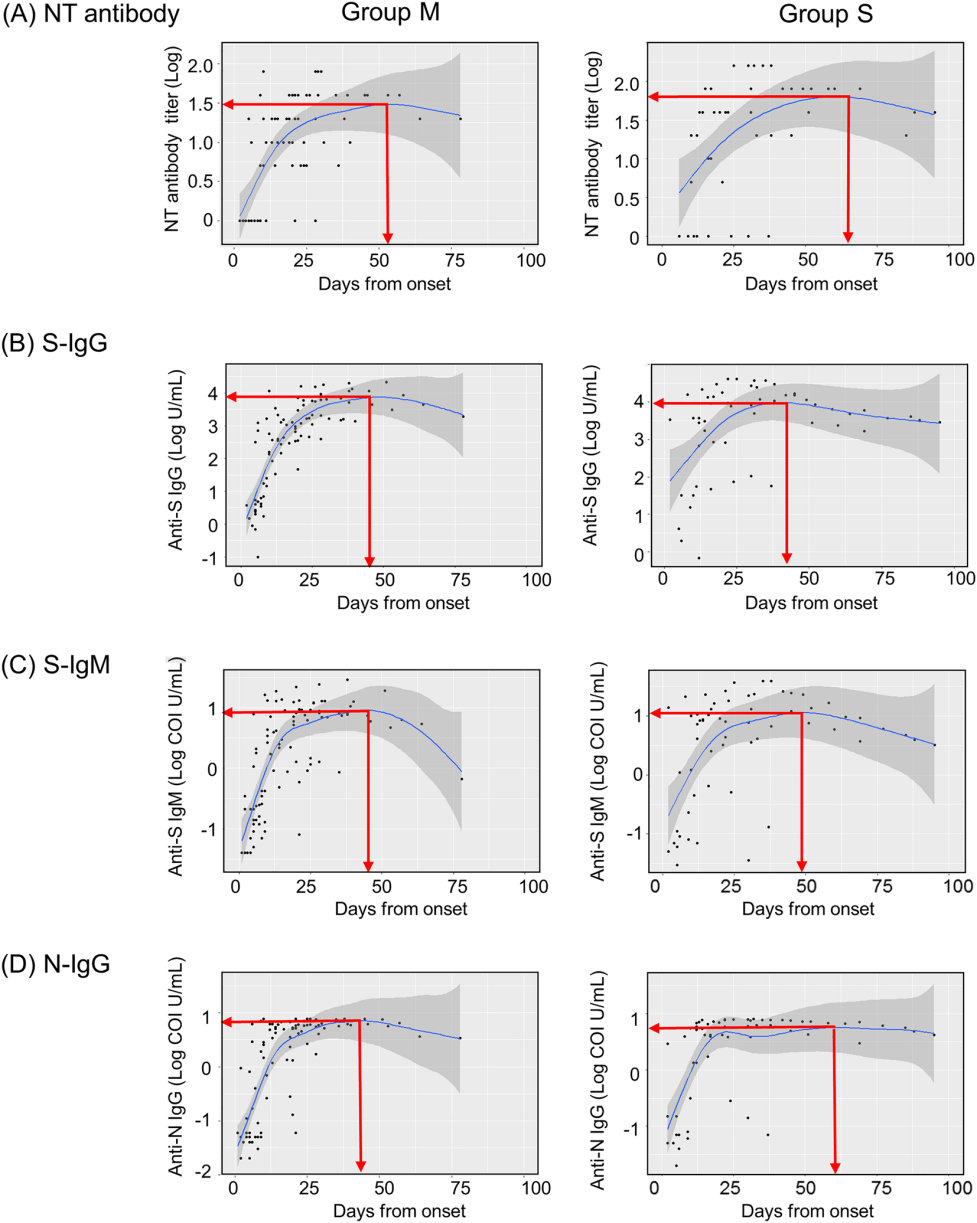
Longitudinal change of antibodies against SARS-CoV-2. Scatterplot and regression lines indicate antibody response for longitudinal analysis: Group M (n = 95 from 52 patients) and Group S (n = 73 from 16 patients). (**A**) NT antibody, (**B**) S-IgG, (**C**) S-IgM, and (**D**) N-IgG. The 95% CIs are calculated by prediction ± 1.96 × standard error of prediction. The red lines indicate the points in which the fitted curves are at their maximum. These points are as follows: (**A**) NT antibody; Group M, day 53, log titer 1.48; Group S, day 69, 1.79, (**B**) S-IgG; Group M, day 46, 3.87; Group S, day 45, 3.96, (**C**) S-IgM; Group M, day 45, 0.96; Group S, day 49, 1.06, (**D**) N-IgG, Group M, day 46, 0.90, Group S, day 60, 0.75.

We then investigated the kinetics of the NT antibody and anti-S-IgG, anti-S-IgM, and anti-N-IgG antibodies using 168 longitudinally assessed samples from the 68 patients. Although all tested antibodies increased in the early phase of infection, neutralization titers of the patients in the severe group (Group S) achieved maximal responses later than the mild symptomatic cases (Group M). Figure 2 shows that maximal NT antibody activity was achieved 53 days after onset (1.48 logs titer) for Group M, while 69 days were required for Group S to develop a maximal response (1.79 logs titer). The speeds of reaching maximum levels of S-IgG and S-IgM antibodies were faster than that of NT antibodies (S-IgG: 3.87 logs at day 46 for Group M, 3.96 logs at day 45 for Group S; S-IgM: 0.96 logs at day 45 for Group M, 1.06 logs at day 49 for Group S). The maximal response of N-IgG antibody was achieved later for Group S (day 58, 0.75 logs) than for Group M (day 46, 0.90 logs).

### Longitudinal assessment in antibody titers

**Figure 3:**
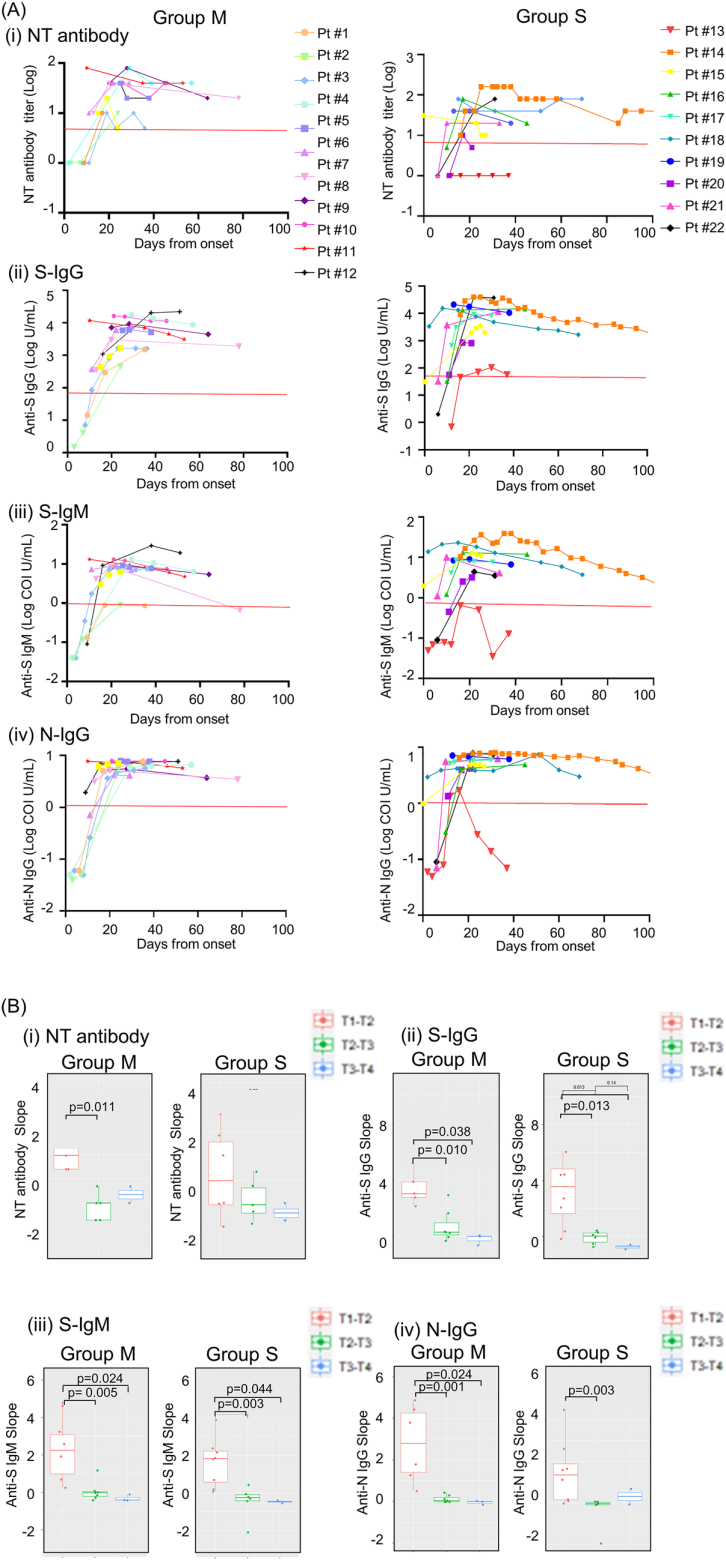
Serial measurements of antibodies against SARS-CoV-2. (**A**) Changes in SARS-CoV-2 antibody titers in 109 serum samples from 22 inpatients measured three or more times in a row were plotted. Group M samples (n = 44 from 12 patients) and Group S samples (n = 65 from 10 patients) were tested. (i) NT antibody, (ii) S-IgG, (iii) S-IgM, and (iv) N-IgG. Clinical characteristics including outcome and past medical history of the patients are shown in Supplementary Table S2. The red horizontal lines indicate the positive threshold of each assay: NT antibody, 5 titer; S-IgG, 50 U/ml; S-IgM, 1.0 COI U/ml; N-IgG, 1.4 COI U/ml. (**B**) Slope analysis of (i) NT antibody, (ii) S-IgG, (iii) S-IgM, and (iv) N-IgG from Group M and Group S. Bars represent the means. Time points are within the following range of days after symptom onset: T1, day 0–13; T2, day 14–27; T3, day 28–41; T4, day 42–55. Statistical significance is indicated as follows: ∗p < 0.05, ∗∗p < 0.01 (Kruskal–Wallis test).

To examine changes in antibody levels over time, we plotted the titers of inpatients measured three or more times in a row (Fig. 3A). A total of 109 samples from 22 cases were collected up to 142 days after symptom onset to determine the change slopes of antibodies, as described in “Materials and methods”. Table 2 summarizes the clinical background characteristics.

**Table 2:** Positivity of SARS-CoV-2 antibody assays.

Severity	GroupM (mild + moderate)	Group S (severe + critical)						
Patient number	52	16						
Sample number	95	73						

Weeks from onset	Sample number	Positive (%)			Sample number	Positive (%)		
		S-IgG	S-IgM	N-IgG		S-IgG	S-IgM	N-IgG
0–6	20	3 (15)	4 (20)	2 (10)	10	1 (10)	3 (30)	1 (10)
7–13	22	11 (50)	11 (50)	10 (45)	11	7 (64)	7 (64)	6 (55)
14–20	16	15 (94)	14 (88)	12 (75)	9	8 (89)	8 (89)	9 (100)
21–27	14	13 (93)	11 (79)	13 (93)	11	11 (100)	10 (91)	10 (91)
28–34	7	8 (100)	8 (100)	8 (100)	7	7 (100)	6 (86)	6 (86)
35–41	4	8 (100)	7 (87.5)	8 (100)	4	4 (100)	3 (75)	3 (75)
42–48	12	12 (100)	12 (100)	12 (100)	21	21 (100)	18 (86)	21 (100)

One severely afflicted patient (Pt #13), who showed suppressed or delayed antibody responses, suffered from liver cancer with cytomegalovirus reactivation and could not be rescued (died after 37 days from onset). In this patient, S-IgG remained positive from day 24 with low titer near the positive threshold line (58.0–105.8 AU/ml). S-IgM was negative throughout the course. N-IgG had a low positive titer on day 16, but N-IgG titer came back negative on day 24. Of the severe cases, Patient #14 was measured for antibodies up to 142 days after onset. S-IgG titers remained high until day 142, while S-IgG and N-IgG decreased after day 127.

To visualize the changes in antibody values, we calculated and plotted the slopes of NT, S-IgG, S-IgM, and S-IgG antibodies based on disease severity (Fig. 3B). Time point 1 (T1), T2, T3 and T4 were within the time frames of 0–13 days, 14–27 days, 28–41 days, and 42–55 days after symptom onset, respectively. From T1 to T2, S-IgG, S-IgM, and N-IgG antibodies increased more rapidly than in the period from T2 to T3 for both Group M and Group S. We observed that NT antibodies increased with a significantly larger slope during T1–T2 than during T2–T3 for Group M, but not for Group S. These findings indicate that in severe cases, NT antibody is activated later than S-IgG, S-IgM, and N-IgG. This is consistent with the result that the timing to obtain maximum NT antibody titer is delayed in Group S, as shown in Fig. 2. No significant difference between Group M and Group S was observed in all tested antibodies among the T1–T2, T2–T3, and T3–T4 slopes.

## Discussion

Although NT antibodies are important for virus clearance and to achieve protection against SARS-CoV-2^22,23^, the kinetics of SARS-CoV-2 antibodies, including NT antibody upon infection, remain controversial.

Concordant with the previous report demonstrating a strong correlation between virus-neutralizing activity and the level of anti-RBD antibodies that block SARS-CoV-2 entry to cells^24^, we observed that levels of S-IgG for RBD of S protein showed a stronger correlation with the NT antibody titers comparing to those of S-IgM and N-IgG. However, this study showed that 15% of the NT antibody negative samples were positive for S-IgG and that the development of NT antibody was slower in comparison to S IgG in severe COVID-19 cases.

These findings indicate that there may be a discrepancy between the antibody levels measured by serologic testing and the neutralizing activity detected by authentic virus neutralization assay. It is known that through a process named affinity maturation, the binding ability of virus-specific IgG antibody is relatively low in the early stage of viral infection and increases with time^25,26^, which is a consequence of B-cells somatic hypermutation. In SARS-CoV-2 infection, anti-RBD antibodies also have been shown to increase in binding affinities and neutralizing potency over time^11,27^. This process might be associated with lower NT antibody activity and a delayed increase compared to S-IgG.

In contrast, Trinité et al. reported a rapid development of NT antibodies in ICU patients and no variations in the kinetic activity between severe and non-severe participants^28^. While the reason for the discrepant findings is not clear, it could be attributed to the different patient conditions examined. Although we included only inpatients in our longitudinal assessment study, Trinité et al. compared hospitalized severe patients to mild symptomatic or asymptomatic patients that were not hospitalized^28^. The methodological difference of NT antibody measurement might be another reason.

It is known that IgM is a potent activator of the classical complement pathway, and plays an important role in the early stages of the immune response and disappears from peripheral blood earlier than IgG^29^. However, in this study, we observed that the seroconversion of anti-SARS-CoV-2 S-IgG and S-IgM antibodies occurred simultaneously, which is concordant with previous reports^30–32^. The seroconversion pattern of SARS-CoV-2 infection suggests the presence of cross-reactive immunity to previously induced general human coronavirus encounters^33^. Furthermore, it has been reported that existing T cell immunity to the common seasonal cold coronaviruses (hCoV-229E, -NL63, -HKU1, and -OC43) can prime the response to SARS-CoV-2^34^.

This study has several limitations. The first is the relatively small number of patients sampled, as well as gender bias within this small sample. Of the 22 patients analyzed, only 4 were female. Because males are known to be more prone to severe disease and higher immune responses due to several possible biological factors, such as hormonal differences and sex-specific genes involved in viral recognition and immune response^35^, it is possible that gender bias could affect the results. Second, while we examined 100 specimens from the pre-COVID-19 period to determine assay specificity, the serological cross-reactivity in samples infected with various corona and influenza viral infections should also be measured. Third, the samples used for determining the kinetics of the specific antibodies, including NT antibody, were mainly obtained from hospitalized COVID-19 patients and did not include asymptomatic cases. Larger cohorts with longer follow-up periods are required, and vaccination further presents an opportunity to elucidate correlates of protection against SARS-CoV-2 infection.

We observed that the quantitative measurement of S-IgG correlated well with the neutralizing activity detected by the neutralization assay. However, these S-IgG antibodies were not completely consistent because their antibody titers are not the same as the neutralization reaction activity.

In conclusion, this study demonstrated the limitation of using the S-IgG antibody titer detected by the chemiluminescent immunoassay as a direct quantitative marker of neutralization activity capacity. Careful interpretation should be given when using antibody test results as markers of serological responses to facilitate diagnosis, treatment, and prophylactic intervention.

## Materials and methods

### Patient cohorts

A total of 168 blood serum samples were collected between March and August 2020 from 68 RT-PCR-confirmed COVID-19 patients who suffered from symptomatic infection. Among them, 22 inpatients (Pt#1–#22), whose serum samples were collected three or more times, have been subjected to longitudinal analysis. Supplementary Table S1 shows the patients’ clinical background.

For the specificity study, 100 serum samples from 100 patients that were collected prior to the emergence of SARS-CoV-2, (2017–2018) were utilized. All samples were obtained from Juntendo University Hospital in Tokyo, Japan. RT-PCR-based molecular testing/confirmation for SARS-CoV-2 was performed using nasopharyngeal specimens by the 2019 Novel Coronavirus Detection Kit (Shimadzu, Kyoto, Japan)^36^. Patients undergoing immunosuppressive drug therapy were excluded from this study^37^.

We first categorized SARS-CoV-2 infection patients as mild, moderate, severe, or critical according to the WHO criteria^21^. Mild COVID-19 was defined as respiratory symptoms without evidence of pneumonia or hypoxia, while moderate or severe infection was defined as the presence of clinical and radiological evidence of pneumonia. In moderate cases, SpO_2_ ≥ 94% was observed on room air, while one of the following was required to identify the severe and critical cases: respiratory rate > 30 breaths/min or SpO_2_ < 94% on room air. Critical illness was defined as respiratory failure, septic shock, and/or multiple organ dysfunction (COVID-19 Clinical management: living guidance. [https://www.who.int/publications/i/item/clinical-management-of-covid-19]). We then stratified them into either Group M, which included mild and moderate cases, or Group S, which included severe and critical cases. Group M patients with a high-risk background were hospitalized and included in the long-term evaluation study.

This study complied with all relevant national regulations and institutional policies. It was conducted in accordance with the tenets of the Declaration of Helsinki and was approved by the Institutional Review Board (IRB) at Juntendo University Hospital (IRB # 20-036). The need for informed consent from individual patients was waived by the Institutional Review Board (IRB) at Juntendo University Hospital because all samples were de-identified in line with the Declaration of Helsinki.

### Neutralization assay

The SARS-CoV-2 ancestral strain WK-521 (lineage A, GISAID ID: EPI_ISL_408667) was used for the authentic virus neutralization assay, which was performed at the National Institute of Infectious Diseases (NIID) with ethics approval by the medical research ethics committee of NIID for the use of human subjects (#1178). The virus neutralization assay was performed as described previously^20^. Briefly, serum samples were serially diluted: twofold serial dilutions starting at 1:5 dilution performed with high glucose Dulbecco's Modified Eagle Medium supplemented with 2% Fetal Bovine Serum (Fujifilm Wako Pure Chemicals, Japan). The diluted samples were mixed with the virus, whose titer in culture supernatant was 6.8 × 10^7^ median tissue culture infectious dose (TCID_50_) per mL at 3 days post infection^38^, and then incubated at 37 °C for 1 h.

The mixture was subsequently incubated with VeroE6/TMPRSS2 cells (JCRB1819, JCRB Cell Bank, Japan) and seeded in 96-well flat-bottom plates for 4–6 days at 37 °C in a chamber supplied with 5% CO_2_. Then the cells were fixed with 20% formalin (Fujifilm Wako Pure Chemicals) and stained with crystal violet solution (Sigma-Aldrich, St Louis, MO, USA). Each sample was assayed in 4–6 wells, and an average cut-off dilution index of ≧ 50% cytopathic effect was presented as neutralization titer. Neutralizing titer of the sample below the detection limit (1:5 dilution) was set as 2.5. Neutralizing (NT) antibody titer of < 5 was considered negative and **≧** 5 was considered positive.

### Serologic testing for SARS-CoV-2

Anti-S SARS-CoV-2 IgG II Quant assay (S-IgG), anti-S SARS-CoV-2 IgM assay (S-IgM), and anti-N SARS-CoV-2 IgG assay (N-IgG) were performed on the Abbott Alinity i platform according to the manufacturer’s instructions. These are the chemiluminescent microparticle (CMIA) assays for quantitative assessment of anti S IgG, semi-quantitative assessment of anti S IgM antibodies, and semi-quantitative assessment of anti N IgG antibodies, respectively. In the S-IgG assay, the SARS-CoV-2 antigen-coated paramagnetic microparticles bind to the IgG antibodies that attach to the receptor binding domain (RBD) of the SARS-CoV-2 spike protein S1 subunit in human serum and plasma samples. The sequence used for the RBD was taken from the WH-Human 1 coronavirus (GenBank accession number MN908947)^39,40^. The S-IgM assay is designed to detect IgM antibody against the whole spike protein, including RBD (https://www.hsa.gov.sg/docs/default-source/hprg-mdb/psar-covid-19-puo-tests/ab03_abbott-alinity-i-sars-cov-2-igm.pdf)^40^. The resulting chemiluminescence in relative light units indicates the strength of the response, which reflects each specific antibody present. Results from the quantitative S-IgG assay are reported as arbitrary units (AU) per milliliter, and values equal to the cutoff of 50 AU/ml or greater were classified as positive^41^. Results from the semi-quantitative anti-S IgM and anti-N IgG assays are reported as index values, and the manufacturer’s suggested positive cutoff points of 1.0 and 1.40 were used, respectively^39,42^.

The clinical linearity of the quantitative anti-S SARS-CoV-2 IgG II Quant assay was evaluated using five patient samples with elevated S-IgG values. Samples were measured at 1:2 dilutions and compared to the theoretical values calculated with their undiluted sample results.

### Statistical analysis

Data analysis was carried out using GraphPad Prism software (version 9.0.1; San Diego, CA, USA) and R software (version 4.1.0). Titers of antibodies were log-transformed before statistical analyses. Correlation analysis between antibody titer and neutralization test was performed using the Spearman correlation coefficient. For the longitudinal analysis, a nonlinear model was fitted for the kinetics of each SARS-CoV-2 antibody. The 95% confidence interval was calculated by performing bootstrap resampling. We conducted a decay analysis of each tested antibody between T1–T2, T2–T3, and T3–T4 for Group M and Group S^20^.

The NT antibodies were log-transformed and the mean of the NT slopes for each patient at each time point (T1–T2, T2–T3, T3–T4) was plotted to visualize the increasing rate of the antibodies of each group. The rate of increase was examined by the Wilcoxon rank-sum test (Fig. 3B). *P* values of < 0.05 were considered statistically significant.

## Supplementary Information

Supplementary Figure S1.

Supplementary Tables.

## Data Availability

All data generated or analyzed during this study are included in this published article and its supplementary information files.
