# Supplementary figures and images for: Activation of SARS-CoV-2 neutralizing antibody is slower than elevation of spike-specific IgG, IgM, and nucleocapsid-specific IgG antibodies
Source: Sci Rep. 2022 Sep 1;12:14909. doi: 10.1038/s41598-022-19073-z (PMC9436163; doi:10.1038/s41598-022-19073-z)

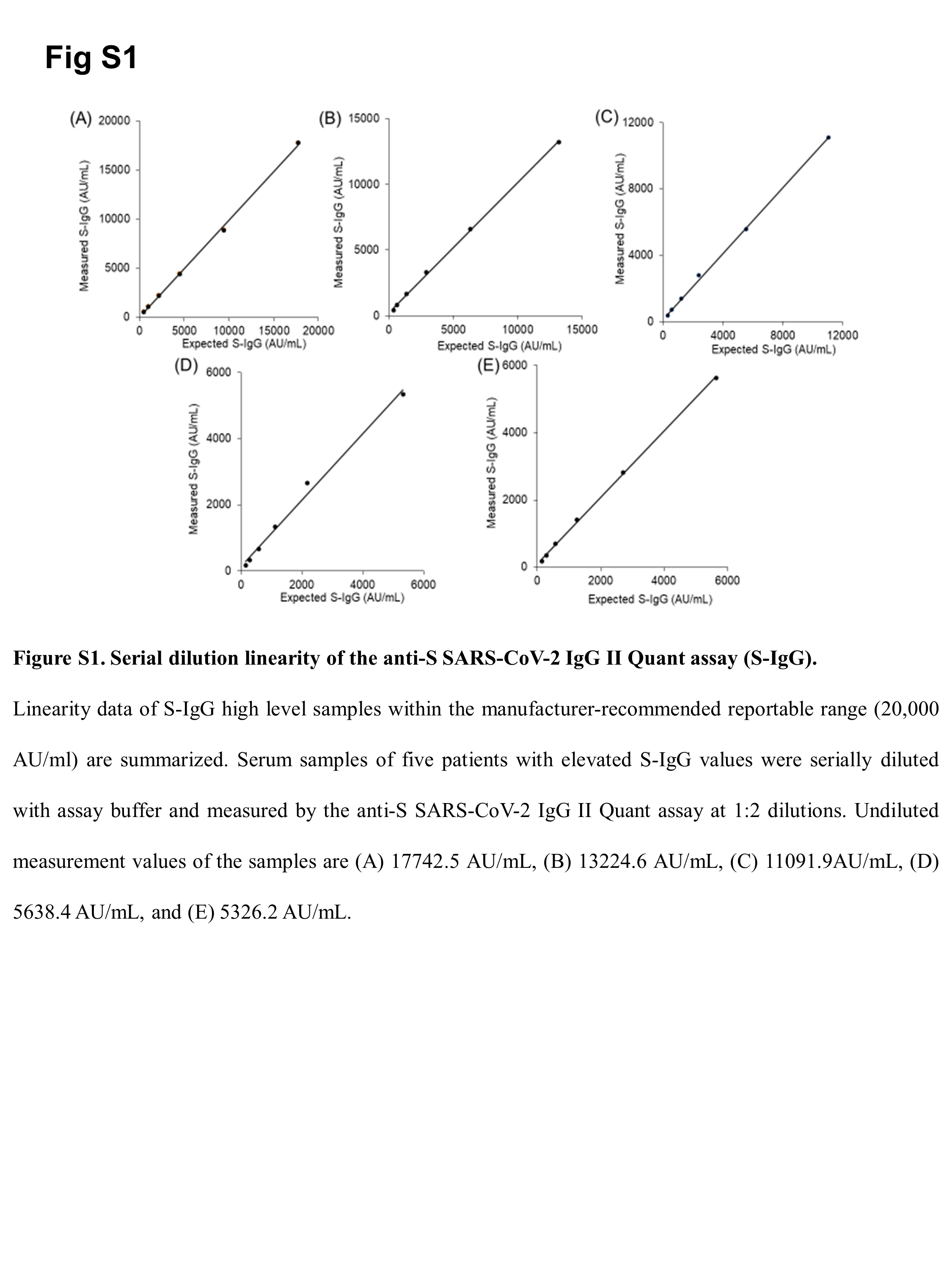

Supplement: Supplementary file 1 — Supplementary Figure S1. [file 41598_2022_19073_MOESM1_ESM.tif]
