# Supplementary material for: Activation of SARS-CoV-2 neutralizing antibody is slower than elevation of spike-specific IgG, IgM, and nucleocapsid-specific IgG antibodies
Source: Sci Rep. 2022 Sep 1;12:14909. doi: 10.1038/s41598-022-19073-z (PMC9436163; doi:10.1038/s41598-022-19073-z)
Supplement: Supplementary file 2 — Supplementary Tables. [file 41598_2022_19073_MOESM2_ESM.docx]

Table S1. Clinical background of patients

| Pt No. | Disease severity | Severity Group | Age* | Sex | Onset time | Epidemic wave** |
| --- | --- | --- | --- | --- | --- | --- |
| 1 | mild | M | 72 | M | 2020/8 | II |
| 2 | moderate | M | 39 | M | 2020/5 | I |
| 3 | moderate | M | 18 | F | 2020/8 | II |
| 4 | mild | M | 84 | M | 2020/7 | II |
| 5 | moderate | M | 75 | M | 2020/4 | I |
| 6 | mild | M | 62 | M | 2020/8 | II |
| 7 | moderate | M | 64 | M | 2020/7 | II |
| 8 | moderate | M | 36 | M | 2020/4 | I |
| 9 | moderate | M | 57 | M | 2020/4 | I |
| 10 | moderate | M | 70 | F | 2020/4 | I |
| 11 | moderate | M | 73 | F | 2020/5 | I |
| 12 | mild | M | 82 | M | 2020/7 | II |
| 13 | critical | S | 83 | M | 2020/7 | II |
| 14 | critical | S | 75 | M | 2020/4 | I |
| 15 | critical | S | 66 | M | 2020/4 | I |
| 16 | severe | S | 76 | M | 2020/4 | I |
| 17 | severe | S | 70 | M | 2020/4 | I |
| 18 | severe | S | 76 | M | 2020/5 | I |
| 19 | severe | S | 14 | M | 2020/4 | I |
| 20 | severe | S | 46 | M | 2020/8 | II |
| 21 | severe | S | 60 | M | 2020/8 | II |
| 22 | severe | S | 79 | F | 2020/7 | II |
| 23 | mild | M | 62 | M | 2020/3 | I |
| 24 | mild | M | 26 | M | 2020/8 | II |
| 25 | mild | M | 74 | F | 2020/6 | II |
| 26 | mild | M | 84 | M | 2020/4 | I |
| 27 | mild | M | 37 | M | 2020/4 | I |
| 28 | mild | M | 36 | M | 2020/6 | II |
| 29 | mild | M | 26 | F | 2020/4 | I |
| 30 | mild | M | 36 | F | 2020/6 | II |
| 31 | mild | M | 50 | M | 2020/4 | I |
| 32 | mild | M | 57 | F | 2020/7 | II |
| 33 | mild | M | 48 | M | 2020/8 | II |
| 34 | mild | M | 29 | M | 2020/7 | II |
| 35 | mild | M | 60 | F | 2020/4 | I |
| 36 | mild | M | 23 | F | 2020/7 | II |
| 37 | mild | M | 31 | F | 2020/7 | II |

| Pt No. | Disease severity | Severity Group | Age* | Sex | Onset time | Epidemic wave** |
| --- | --- | --- | --- | --- | --- | --- |
| 38 | mild | M | 26 | M | 2020/7 | II |
| 39 | mild | M | 53 | F | 2020/4 | I |
| 40 | mild | M | 60 | M | 2020/4 | I |
| 41 | mild | M | 52 | M | 2020/7 | II |
| 42 | mild | M | 34 | M | 2020/3 | I |
| 43 | mild | M | 33 | F | 2020/5 | I |
| 44 | mild | M | 28 | M | 2020/4 | I |
| 45 | mild | M | 42 | M | 2020/6 | II |
| 46 | mild | M | 36 | F | 2020/7 | II |
| 47 | mild | M | 29 | M | 2020/7 | II |
| 48 | mild | M | 36 | M | 2020/6 | II |
| 49 | mild | M | 30 | M | 2020/8 | II |
| 50 | mild | M | 31 | M | 2020/8 | II |
| 51 | moderate | M | 74 | F | 2020/8 | II |
| 52 | moderate | M | 57 | M | 2020/7 | II |
| 53 | moderate | M | 52 | F | 2020/8 | II |
| 54 | moderate | M | 48 | M | 2020/8 | II |
| 55 | moderate | M | 48 | M | 2020/8 | II |
| 56 | moderate | M | 29 | M | 2020/7 | II |
| 57 | moderate | M | 47 | M | 2020/7 | II |
| 58 | moderate | M | 40 | F | 2020/8 | II |
| 59 | moderate | M | 45 | M | 2020/8 | II |
| 60 | moderate | M | 32 | F | 2020/8 | II |
| 61 | moderate | M | 53 | M | 2020/8 | II |
| 62 | moderate | M | 37 | F | 2020/8 | II |
| 63 | severe | S | 53 | M | 2020/8 | II |
| 64 | severe | S | 56 | M | 2020/7 | II |
| 65 | severe | S | 58 | M | 2020/8 | II |
| 66 | critical | S | 88 | M | 2020/8 | II |
| 67 | critical | S | 78 | F | 2020/7 | II |
| 68 | critical | S | 79 | M | 2020/7 | II |

*average age Group M 61.0

y.o. (14 – 79) and Group S 64.5 y.o. (18 – 84), p=0.694

** The predominant lineage of the first wave, B.1.1; second wave, B.1.1.284

Table S2. Clinical characteristics of patients longitudinally analyzed

| Pt No. | Outcome | Past Medical history |
| --- | --- | --- |
| 1 | cure/discharge | No known |
| 2 | cure/discharge | Pulmonary sarcoidosis |
| 3 | cure/discharge | No known |
| 4 | cure/discharge | Colon cancer, Parkinson's disease, Dementia |
| 5 | cure/discharge | Benign prostatic hyperplasia |
| 6 | cure/discharge | No known |
| 7 | cure/discharge | Postoperative pancreatic cancer |
| 8 | cure/discharge | No known |
| 9 | cure/discharge | Hypertension, DL |
| 10 | cure/discharge | DL, DM |
| 11 | cure/discharge | Hypertension, DL, AP |
| 12 | cure/discharge | Cholangiocarcinoma |
| 13 | dead | HCC, CKD |
| 14 | dead | DM, HT, Prostatic cancer |
| 15 | dead | Diabetic Nephropathy |
| 16 | cure/discharge | None |
| 17 | cure/discharge | Gastric polyp |
| 18 | cure/discharge | DM, RA, PD, PDD |
| 19 | cure/discharge | Kidney stones, Alcoholic liver disease |
| 20 | cure/discharge | T2DM, Stiff-person syndrome, Angina, DL |
| 21 | cure/discharge | Angina, HT, PSVT |
| 22 | cure/discharge | HT, Asymptomatic cerebral infarction |

DL, Dyslipidemia; DM, Diabetes mellitus; AP, Angina pectoris; RA, rheumatoid arthritis; PD, Progressive disease;

PDD, Pervasive developmental disorder; HT, Hypertension; PSVT, Paroxysmal supraventricular tachycardia;

HCC, hepatocellular carcinoma; CKD, chronic kidney disease
